# Integrating Physiology Into Hyponatremia Algorithms: Moving Beyond Volume Status

**DOI:** 10.1177/23247096261474690

**Published:** 2026-07-29

**Authors:** Jodie Mogensen, Livia Frost, Bryan Tucker, L. Parker Gregg, Maulin K. Shah

**Affiliations:** 1 Department of Internal Medicine, 3989 Baylor College of Medicine, Houston, TX, USA; 2 School of Medicine, 3989 Baylor College of Medicine, Houston, TX, USA; 3 Department of Medicine, Selzman Institute for Kidney Health, 3989 Baylor College of Medicine, Houston, TX, USA; 4 Veterans Affairs Health Services Research & Development Center for Innovations in Quality, Effectiveness, and Safety, Houston, TX, USA; 5 Division of Nephrology, Michael E DeBakey VA Medical Center, Houston, TX, USA

**Keywords:** hyponatremia, diagnostic reasoning, diagnostic error, volume status, tonicity

## Abstract

Hyponatremia is a prevalent electrolyte disorder with a broad differential diagnosis, which can pose a challenge for determining the underlying etiology. Academic resources teach two diagnostic frameworks designed to guide a physician to the underlying etiology. One approach uses volume status and serum osmolality as earlier branch points, with later consideration of urine osmolality and urine sodium for diagnosis within certain pathways in the branching logic. The other uses serum osmolality, urine osmolality, and spot urine sodium to guide the entire diagnostic reasoning process. These two approaches can ultimately guide clinicians to different diagnoses for the same patient. In this case series, we present an adapted physiology-based (European) algorithm and describe three patients who illustrate key shortcomings of the volume status-based approach to identifying the etiology of hyponatremia. The first case emphasizes that understanding the relationship between osmolality and tonicity adds important nuance to the interpretation of measured serum osmolality. The second case demonstrates how the inherent limitations of the physical examination for volume status contribute to imprecision and inter-individual variation in volume status assessment and underscores the importance of interpreting laboratory values with nuance rather than relying rigidly on strict cutoff values as branch points in clinical reasoning. The third case illustrates that more complex presentations may not fit neatly within any single algorithm and require more nuanced clinical judgment. Together, these cases demonstrate how a diagnostic approach to hyponatremia rooted in physiology provides learners with greater insight into the underlying pathophysiology and may improve diagnostic accuracy.

## Introduction

Hyponatremia is a common electrolyte disorder encountered in both inpatient and outpatient settings. Prompt and accurate determination of the etiology of hyponatremia is imperative to guide management decisions. Unfortunately, the etiology of hyponatremia can be challenging to diagnose.^1^ The broad differential diagnosis, possibility of multiple concomitant mechanisms in medically complex patients, and limitations in algorithmic clinical reasoning may contribute to diagnostic error.^1,2^ There are two predominant diagnostic algorithms used clinically to determine of the etiology of hyponatremia. The first approach, favored in the U.S. guideline, involves earlier diagnostic branch points based on serum osmolality and assessment of volume status, with urine osmolality and urine sodium used to guide the differential diagnosis within certain downstream pathways (hereafter referred to as the volume status-based approach).^3^ While conceptually straightforward, this framework has important limitations that may guide the clinician to the incorrect diagnosis.^2^ The second approach, favored in the European guideline, reframes the diagnostic reasoning process to emphasize urine osmolality and spot urine sodium concentrations as representative of hormonal activity (hereafter referred to as the physiology-based approach).^4^ These two clinical reasoning schema guide the clinician through the diagnostic process in different ways and may yield different diagnoses.

**Figure 1.**
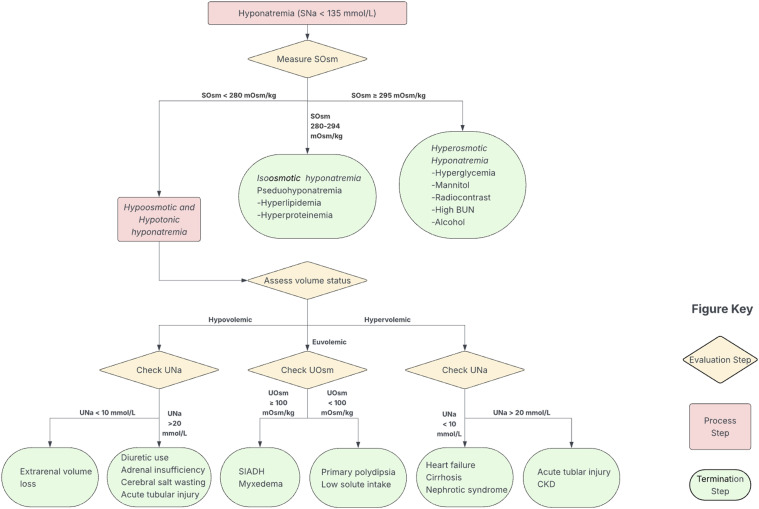
Evaluation of hyponatremia using a volume status-based approach (U.S. guideline). Abbreviations: ADH, antidiuretic hormone; CKD, chronic kidney disease; Osm gap, osmolal gap; RAAS, renin-angiotensin-aldosterone system; SIADH, syndrome of inappropriate antidiuretic hormone secretion; SNa, serum sodium; SOsm, serum osmolality; UNa, urine sodium; UOsm, urine osmolality

**Figure 2.**
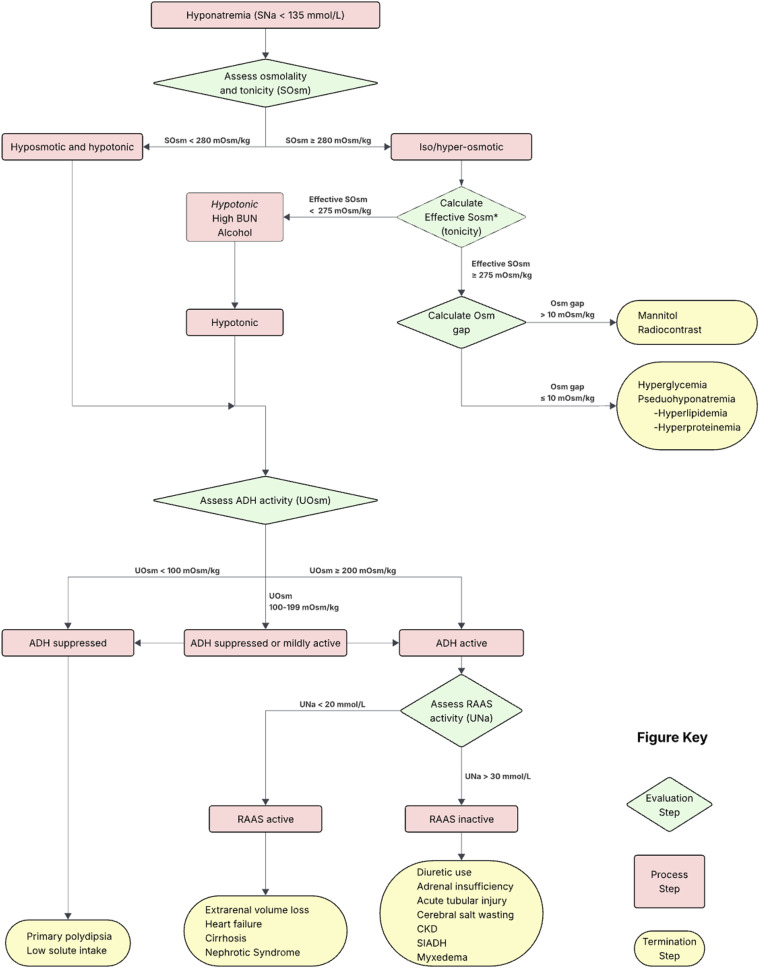
Evaluation of hyponatremia using a physiology-based approach (modified European guideline). Abbreviations: ADH, antidiuretic hormone; CKD, chronic kidney disease; Osm gap, osmolal gap; RAAS, renin-angiotensin-aldosterone system; SIADH, syndrome of inappropriate antidiuretic hormone secretion; SNa, serum sodium; SOsm, serum osmolality; UNa, urine sodium; UOsm, urine osmolality *Effective serum osmolality is calculated as follows: $Measured\;SOsm-\frac{BUN}{2.8}-\frac{EtOH}{4.6}$

Here, we present three clinical cases of chronic hyponatremia that illustrate some ways in which the volume status-based (U.S. guideline) approach (Figure 1) may arrive at an incorrect diagnosis. Specifically, these cases highlight that basing clinical reasoning on strict cutoffs of laboratory values, reliance on subjective assessments, and poor representation of underlying physiology may all contribute to error. We present an adapted physiology-based (European guideline) algorithm (Figure 2) to demonstrate how an alternative approach that is more strongly rooted in pathophysiology can circumvent these pitfalls.

### Case 1: Understanding the Relationship Between Osmolality and Tonicity

A 66-year-old man with a history of hypertension, squamous cell carcinoma at the base of the tongue treated with chemotherapy and radiation 3 years previously, prostate cancer treated with radical prostatectomy and lymph node dissection 10 years previously, remote deep vein thrombosis, and liver cirrhosis presented to primary care clinic with a seven-year history of chronic moderate hyponatremia. Seven years previously he had presented with syncopal episodes. He was found to have orthostatic hypotension treated with intravenous fluids. Subsequent work-up by the admitting team revealed hypotonic hyponatremia with a serum sodium of 123 mmol/L, serum osmolality of 270 mOsm/kg, a urine sodium of 43 mmol/L, and a urine osmolality of 286 mOsm/kg. His hyponatremia was attributed to hypovolemia with serum sodium normalizing to 140 mmol/L on follow-up from the discharge value of 124 mmol/L. Hyponatremia recurred seven months after discharge, and he had intermittent hyponatremia until three years prior to the current presentation, when his sodium levels decreased and remained consistently between 121 and 127 mmol/L until the current presentation. By the time of the current presentation, he had been diagnosed with cirrhosis and treated for squamous cell carcinoma. On physical examination, he appeared euvolemic, with no edema, pulmonary rales, jugular venous distention, or signs of hypovolemia. His neurologic examination was unremarkable. His laboratory values at presentation are displayed in Table 1. Lipid panel and serum protein levels were normal one month previously with a concurrent sodium level of 125 mmol/L.

**Table 1.** Case 1 Laboratory Values at Presentation

Laboratory test	Result	Reference range
Sodium	126 mmol/L	136-145 mmol/L
Potassium	4.2 mmol/L	3.6-5.0 mmol/L
Chloride	90 mmol/L	98-107 mmol/L
Bicarbonate	21 mmol/L	22-30 mmol/L
Blood Urea Nitrogen (BUN)	7 mg/dL	7-19 mg/dL
Creatinine	0.85 mg/dL	0.6-1.3 mg/dL
Glucose	86 mg/dL	70-110 mg/dL
Albumin	3.1 g/dL	3.5-4.9 g/dL
Ethanol	144 mg/dL	<10 mg/dL
Serum Osmolality	293 mOsm/kg	280-301 mOsm/kg
Urine Osmolality	239 mOsm/kg	None established
Urine Sodium	11 mmol/L	None established

Using the volume status-based approach, a serum osmolality of 293 mOsm/kg suggests isoosmolar hyponatremia (Figure 1). However, hyperlipidemia and paraproteinemia were ruled out as causes that might contribute to elevated osmolality in the setting of multiple concomitant etiologies. Upon further evaluation, his calculated serum osmolality was 259 mOsm/kg. This yielded an osmolal gap of 33, which normalized after accounting for his ethanol level of 144 mg/dL. Therefore, his labs were consistent with hypotonicity despite isoosmolality, which guides clinical reasoning instead toward a differential diagnosis for hypotonic hyponatremia (Figure 2). Given the patient’s risk factors for multiple possible etiologies of hypotonic hyponatremia, particularly low solute intake and poor perfusion in the setting of cirrhosis, additional diagnostic workup was deferred until ethanol levels normalized. Unfortunately, the patient passed away at an outside hospital before further outpatient workup was obtained.

This case illustrates the importance of understanding the difference between osmolality and tonicity. Although these terms are often incorrectly used interchangeably, the difference between them has implications for interpreting the measured serum osmolality. Osmolality refers to the total number of dissolved particles (or osmoles) in one kilogram of solvent (in the case of plasma osmolality, the solvent is plasma water).^5^ Tonicity refers to a subset of osmoles that cannot freely cross the cell membrane and thus induce the net movement of water across the cell membrane to balance total osmolality between the intracellular and extracellular spaces.^5,6^ This movement of water dilutes serum sodium in cases of hypertonic hyponatremia caused by osmoles that cannot freely cross the cell membrane, such as glucose (in the absence of insulin) or mannitol.

However, osmoles that can freely diffuse across the cell membrane such as urea and ethanol do not contribute to tonicity. These are generally referred to as ineffective osmoles (as contrasted to effective osmoles which do contribute to tonicity). Ineffective osmoles do not cause water to move across the cell membrane to dilute the serum sodium concentration and thus do not cause hyponatremia.^5^ To identify the presence of unmeasured osmoles, one can calculate the osmolal gap^7^:

Osmolal gap=Measured serum osmolality−Calculated serum osmolality

$$Osmolal\;gap=Measured\;serum\;osmolality-\left(2\;x\;sodium+\frac{glucose}{18}+\frac{BUN}{2.8}\right)$$

The osmolal gap is considered elevated if it is >10 mOsm/kg. In cases with an elevated osmolal gap, the effective serum osmolality (i.e., tonicity) can be calculated by accounting for commonly elevated ineffective osmoles (urea and alcohol)^8-11^ by the following equation:

$$Effective\;serum\;osmolality=Measured\;serum\;osmolality-\frac{BUN}{2.8}-\frac{Ethanol\;level}{4.6}$$

In this case, by dividing the measured serum ethanol concentration by 4.6 (due to its molecular weight of 46 g/mol)^11^ we find that ethanol was contributing approximately 31 ineffective osmoles to the total measured serum osmolality. Therefore, despite a normal measured serum osmolality, this patient had hypotonic hyponatremia, which had significant implications for the differential diagnosis.

Additionally, although this patient initially was thought to have presented with hypovolemic hyponatremia, his urine sodium of 43 mmol/L could have misleadingly suggested inadequate aldosterone activity. This is likely attributable to the fact that his urine sample was obtained after he received intravenous fluids, which would have suppressed aldosterone and altered the expected laboratory profile. This case underscores how urine studies are a window into the prevailing hormonal and physiologic state *at the time of collection*. Interpreting laboratory results strictly according to the algorithm assumes that they were obtained prior to therapeutic interventions. However, in the real-world clinical setting it is not always possible to collect a sample prior to the delivery of urgent interventions. Because therapeutic interventions can rapidly modify hormonal activity, understanding the underlying physiology and how those interventions would be expected to alter the hormonal milieu allows the clinician to interpret laboratory values that may not cleanly align with the algorithm.

#### Case 1 Teaching Points

Not all measured osmoles contribute to tonicity. An elevated gap between the calculated and measured osmoles can point to the presence of unmeasured osmoles, which may be effective or ineffective and impact the interpretation of the measured serum osmolality as it pertains to tonicity. Understanding the difference between osmolality and tonicity can guide the clinician down a different diagnostic thought process than algorithmically following strict cutoffs of the measured serum osmolality. Interventions can also alter laboratory values, so clinicians must account for treatment effects when interpreting results.

### Case 2: Reasoning by Objective Measurements

A 68-year-old man with chronic hyponatremia of 120-130 mmol/L for ten years, hypertension, cerebrovascular accident complicated by seizures, neurosyphilis, and severe alcohol use disorder presented with progressive confusion, falls, and poor oral intake. According to his family, his confusion began one year previously but noticeably worsened over the past month. On admission, his serum sodium level was 114 mmol/L. On physical examination he was noted to have distended jugular veins and trace peripheral edema, but lungs were clear to auscultation. His laboratory values at presentation are displayed in Table 2.

**Table 2.** Case 2 Laboratory Values at Presentation

Laboratory test	Result	Reference range
Sodium	114 mmol/L	136-145 mmol/L
Potassium	2.8 mmol/L	3.6-5.0 mmol/L
Chloride	93 mmol/L	98-107 mmol/L
Bicarbonate	26 mmol/L	22-30 mmol/L
BUN	6 mg/dL	7-19 mg/dL
Creatinine	1.6 mg/dL	0.7-1.3 mg/dL
Glucose	98 mg/dL	70-110 mg/dL
Albumin	2.6 g/dL	3.5-4.9 g/dL
Brain natriuretic peptide (BNP)	65 pg/mL	<100 pg/mL
Ethanol	88 mg/dL	<10 mg/dL
Serum Osmolality	257 mOsm/kg	280-301 mOsm/kg
Urine Osmolality	101 mOsm/kg	None established
Urine Sodium	<10 mmol/L	40-220 mmol/L
Urine Specific Gravity	1.005	1.005-1.030

A chest radiograph showed no acute cardiopulmonary findings. Transthoracic echocardiogram revealed no pericardial effusion and normal left ventricular systolic function with impaired relaxation and normal filling pressures. Assessment of the right heart was limited, but the right ventricle appeared enlarged with normal systolic function. Right atrium and pulmonary artery systolic pressures could not be estimated due to poor acoustic windows. Inferior vena cava collapsibility was not mentioned in the report.

After establishing hypoosmolality, the volume status-based diagnostic algorithm relies on the physical examination of the volume status to guide the next step in clinical reasoning. Based on the presence of distended jugular veins and peripheral edema, a volume status-based clinical reasoning algorithm may guide a clinician to diagnose hypervolemic hyponatremia in this case (Figure 1). A more objective and physiologic approach bypasses volume status as its first diagnostic branchpoint, as it is more prone to error, and instead uses the urine osmolality to determine whether antidiuretic hormone (ADH) activity caused water retention in the nephron, leading to hyponatremia (Figure 2). In the setting of hypotonic hyponatremia, a low urine osmolality indicates maximally dilute urine and low ADH activity, whereas a higher urine osmolality indicates the presence of ADH reabsorbing water from the distal nephron and leading to a more concentrated urine. In elevated ADH states, the spot urine sodium concentration can be used to indicate whether the renin-angiotensin-aldosterone system (RAAS) is activated. A low urine sodium indicates that the kidneys are reabsorbing almost all the filtered sodium, which indicates that RAAS is being stimulated, typically by hypoperfusion. One notable exception to this is that hypoperfusion due to profuse vomiting can present with a relatively higher urine sodium and low urine chloride. Another caveat to interpreting these values is that they can be affected by the use of certain medications such as diuretics or angiotensin II receptor blockers, so careful review of the patient’s medications and consideration of their effects is a key component of the diagnostic assessment. In this case, a low urine osmolality of 101 mOsm/kg indicates that ADH was suppressed, and the urine was dilute. In the context of the patient’s clinical history of drinking more than 24 beers per day and poor oral intake, he was diagnosed with non-ADH mediated hypotonic hyponatremia due to low solute intake and high fluid intake (beer potomania). He was successfully managed with combination of hypertonic saline and scheduled desmopressin.

The physical examination for volume status is neither sensitive nor specific for identifying states of intravascular volume excess or depletion and can be poorly indicative of the effective circulating volume.^12-14^ For example, peripheral edema may be present without total-body hypervolemia. As seen in our patient, low solute intake (beer potomania) is associated with malnutrition and hypoalbuminemia, which can contribute to peripheral edema due to decreased oncotic pressure and impaired lymphatic return.^15^ Another common example is distributive shock from sepsis, in which microvascular endothelial permeability contributes to the development of peripheral edema despite intravascular volume depletion.^16^ Similarly, few physical examination findings other than frank orthostasis accurately identify hypovolemic states.^17^ Centering clinical reasoning on objective measurements that reflect the effective circulating volume and end-organ perfusion rather than insensitive and nonspecific physical examination findings can lead the clinician to more clearly understand the pathophysiology and arrive at the correct diagnosis of the underlying etiology of hyponatremia.

Furthermore, using rigid cutoffs for interpreting laboratory values, such as a urine osmolality cutoff of 100 mOsm/kg to distinguish dilute or concentrated urine, may lead the clinician down an inappropriately narrow thought process. In this case, even if determining that the patient was euvolemic, this patient’s urine osmolality might lead one to a differential diagnosis of syndrome of inappropriate ADH (SIADH) or myxedema rather than primary polydipsia or low solute intake, despite that a urine osmolality of 101 mOsm/kg indicates dilute urine. A more flexible interpretation of such laboratory value cutoffs becomes particularly important in people with reduced diluting capacity, such as older adults, people on thiazide diuretic therapy, or people with chronic kidney disease.^1,18^ Such patients may have a higher minimum achievable urine osmolality than would a healthy person.^1^ Consequently, while a urine osmolality <100 mOsm/kg unambiguously indicates maximally dilute urine, considering a “gray zone” of a urine osmolality between 100-200 mOsm/kg that may include either mildly elevated ADH states or ADH-suppressed states in the differential diagnosis may help clinicians think physiologically and avoid diagnostic error.

#### Case 2 Teaching Points

Because the physical examination for volume status is inherently insensitive and nonspecific, clinical reasoning that depends exclusively on this can be prone to error. Instead, using objective assessments such as the urine osmolality to indicate the activity of ADH and the urine sodium to indicate the activity of the RAAS system can lead to more accurate diagnosis. Interpreting laboratory values in the context of the patient’s underlying physiology rather than strictly following cutoff values can help maintain a more inclusive differential diagnosis, particularly in borderline cases.

### Case 3: Thinking Physiologically When the Case doesn’t Fit the Algorithm

A 76-year-old man with a history of hypertension and chronic kidney disease was transferred from an outside center for the management of metastatic gallbladder cancer. He was found to have ascites secondary to hepatic dysfunction from liver metastases. Imaging revealed dilated pulmonary arteries on chest radiograph and dilated left and right atria on transthoracic echocardiogram. He was initiated on furosemide.

During admission, he had mild hyponatremia ranging from 129 to 131 mmol/L, which failed to improve with diuresis. Isotonic intravenous fluids were administered, which caused the sodium concentration to decrease to 124 mmol/L, prompting a nephrology consultation. On nephrology’s physical examination, he was confused and disoriented, which was a departure from his baseline mental status. He had distended jugular veins and pitting edema of the lower extremities. His laboratory values at the time of nephrology consultation are displayed in Table 3.

**Table 3.** Case 3 Laboratory Values at the Time of Nephrology Consultation

Laboratory test	Result	Reference range
Sodium	124 mmol/L	136-145 mmol/L
Potassium	4.0 mmol/L	3.6-5.0 mmol/L
Chloride	88 mmol/L	98-107 mmol/L
Bicarbonate	21 mmol/L	22-30 mmol/L
Creatinine	1.28 mg/dL	0.7-1.3 mg/dL
Blood Urea Nitrogen (BUN)	15 mg/dL	7-19 mg/dL
Glucose	124 mg/dL	70-110 mg/dL
TSH	4.5 mlU/L	0.5-5.0 mIU/L
Serum Osmolality	263 mOsm/kg	280-301 mOsm/kg
Urine Osmolality	430 mOsm/kg	None established
Urine Sodium	<10 mmol/L	None established
Casts	Hyaline casts present	None

Although the volume status-based algorithm (Figure 1) and the physiology-based algorithm (Figure 2) lead to overlapping differential diagnoses, the listed etiologies do not perfectly fit this patient’s clinical scenario. This case illustrates the importance of incorporating the entire clinical picture into the diagnostic reasoning process when patients do not neatly fit into the algorithmic differential diagnosis. The urine osmolality and urine sodium indicate that ADH was elevated and the RAAS system was activated; hence, hypoperfusion was appropriately stimulating ADH secretion. The presence of hyaline casts on urinalysis further supports this. Multiple causes of decreased effective circulating volume may contribute to hypoperfusion, including hypovolemic states such as sepsis or hemorrhage, or hypervolemic states such as heart failure or cirrhosis. Although this patient did not have a history of heart failure, his dilated atria and elevated pulmonary artery pressures on echocardiogram suggested chronic pressure and volume overload. He also did not have a formal diagnosis of cirrhosis, but he did have metastases to the liver and peritoneum with moderate ascites, indicating third-spacing of fluid. The patient’s serum sodium level decreased after the administration of isotonic crystalloid, which further aids in diagnostic reasoning, suggesting that the elevated-ADH state (and thus the hypoperfusion) was not alleviated by crystalloid. In light of the patient’s entire clinical picture incorporating history, physical examination, imaging, laboratory results, and response to treatments, the hyponatremia was ultimately attributed to decreased effective circulating volume in the setting of decompensated heart failure and metastatic malignancy. His sodium gradually improved with more aggressive diuresis. Unfortunately, given the advanced stage of his gallbladder carcinoma, he soon transitioned to comfort care and passed away.

Hyponatremia often occurs in medically complex scenarios in which multiple (sometimes competing) underlying factors may be at play and a single diagnosis cannot alone explain the clinical results. In this case, the volume status-based algorithm led to a reasonable differential diagnosis of hypervolemic hyponatremia. However, without a more detailed physiologic understanding of what the laboratory results indicated about how the kidneys were perceiving end-organ perfusion, the listed entities in this algorithm did not fully explain the patient’s presentation. The initial step of diagnostic reasoning was rooted in the concept that decreased effective circulating volume is a potent non-osmotic stimulus for ADH secretion, although it was initially unclear whether this was primarily a function of hypovolemia related to ascites and third spacing of fluid or of venous congestion and decompensated heart failure. A better understanding of the underlying physiology combined with a comprehensive assessment of the patient’s clinical picture (beyond volume status alone) is crucial to reason through complex cases that do not necessarily neatly fit into a single diagnosis or that have multiple concurrent factors contributing to hyponatremia.

Another important caveat is that the patient received diuretics prior to obtaining key laboratory tests. In this case, given the kidneys were hypo-perfused from decreased effective circulating volume, RAAS was activated and the urine sodium was low at the time of measurement. However, receipt of diuretics can iatrogenically increase the urine sodium concentration independent of RAAS activity. In such cases, the fractional excretion or urea and uric acid can be used as alternative markers of RAAS activation. RAAS activation causes increased urea resorption in the inner medullary collecting ducts and increased uric acid reabsorption in the proximal tubules. Consequently, low fractional excretion of urea and uric acid are valid in the setting of diuretic use and can help the clinician determine the underlying physiology.^13,18-20^

#### Case 3 Teaching Point

Although algorithms can be useful, particularly for learners, no algorithm will identify the correct diagnosis with perfect accuracy. A diagnostic reasoning framework based in the underlying pathophysiology can help the clinician reason through more complex cases when the algorithm is inadequate to fully explain the clinical picture. Incorporating the entirety of the patient’s data, including the history, physical examination, medications, laboratory values, imaging findings, and response to prior treatments, is crucial for complex clinical reasoning and provides a great deal of informative context beyond the physical examination alone.

## Conclusions

The etiology of chronic hyponatremia has a complex differential diagnosis and can be challenging to diagnose. A diagnostic reasoning framework for hyponatremia that is based in physiology and objective assessments overcomes important limitations of the volume status-based algorithm and may help reduce diagnostic error. Understanding the relationship between tonicity and osmolality, using objective rather than subjective assessments, understanding exceptions and gray areas to laboratory value interpretation, and considering the underlying physiology in the context of the whole clinical picture can improve diagnostic accuracy in complex cases.
